# A Case of Perforating Ulcer of the Stomach; Recovery

**Published:** 1871

**Authors:** T. Tinley


*A Case of Perforating Ulcer of the Stomach; Recovery.* By T. Tinley, L.R.C.P., Edin., etc.

I am induced to forward the particulars of the following case of recovery after perforation of the stomach, it being similar to the one recorded by Dr. Ross, occurring in North Staffordshire

Infirmary, and published in the *Lancet* of May. As I have not any notes of the case, I have to rely entirely on my memory, and can only give the line of treatment pursued, regardless of dates.

I was sent for on November 21st, 1868, to visit Mrs. B——, who had been taken suddenly ill. On arriving at the house, I found her in a state of collapse, with a quick, scarcely perceptible pulse, pallid, and covered with cold, clammy perspiration. There was constant retching, but no vomiting of any account; merely a small quantity of grumous fluid, having the appearance of blood acted on by the gastric juice. I remained with her some time, and gave her a dose of opium. I found, on inquiry, that for some time she had complained of a burning pain coming on shortly after taking food. The pain was at a fixed point in the epigastric region, shooting through to the back, accompanied by nausea, but only occasional vomiting. She had never sought relief, thinking it would get better. On the morning of the 21st, while preparing dinner, she was suddenly seized with severe pain in the epigastrum, causing her to scream violently, which she continued to do at intervals for some time. Fomentations were applied to the abdomen constantly, and a grain of opium given in pill every third hour, which procured very little rest, and only partially relieved the pain.

On the second day after the occurrence of the above symptoms, the pain had extended over the whole abdomen; the knees were drawn up, the pulse became very high and wiry, the countenance anxious, and, in fact, all the symptoms of acute general peritonitis set in. I ordered a turpentine stupe to the abdomen, to be followed by hot fomentations; and beef-tea and brandy injections every fourth hour. Neither liquid nor solid was taken into the stomach for above a week. Her thirst was relieved by gargling with cold water. The stupe and fomentations relieved her to a considerable extent, and she gradually but slowly improved; but up to December 11th, the abdomen remained tympanic and painful on pressure. I then ordered a blister to be applied over the whole abdomen, after which she progressed rapidly, and has been in good health ever since.

I am inclined to look on the above as a clear case of gastric ulcer ending in perforation; for although the symptoms of ulceration had not been so well marked as they sometimes are, still the pain occurring shortly after taking food, aggravated by hot liquids, such as hot tea; the burning character of the pain, shooting through to the back; the sudden occurrence of excruciating pain, first in the epigastric region, followed by general peritonitis; leave little doubt as to the nature of the case. I believe the favorable termination of the case is to be attributed to its occurring at a time (just before a meal) when the stomach was, in all probability, almost if not quite empty, the previous meal—breakfast, being very likely composed of food easily digestible.—*Lancet.*

Whitley, Yorkshire, January 25th, 1871.

